# Supplementary material for: Mangrove reforestation provides greater blue carbon benefit than afforestation for mitigating global climate change
Source: Nat Commun. 2023 Feb 10;14:756. doi: 10.1038/s41467-023-36477-1 (PMC9918466; doi:10.1038/s41467-023-36477-1)
Supplement: Supplementary file 1 — Supplementary Information [file 41467_2023_36477_MOESM1_ESM.pdf]

Supplementary Information for

Mangrove reforestation provides greater blue carbon benefit than afforestation for mitigating global climate change

Shanshan Song[1, 2], Yali Ding[1*], Wei Li[1*], Yuchen Meng[1,2], Jian Zhou[1], Ruikun Gou[1, 2], Conghe Zhang[1, 2], Shengbin Ye[1,2], Neil Saintilan[3], Ken W. Krauss[4], Stephen Crooks[5], Shuguo Lv[6], Guanghui Lin[1, 2*]

[1]Department of Earth System Science, Ministry of Education Key Laboratory for Earth System Modeling, Institute for Global Change Studies, Tsinghua University, Beijing 100084, China.

[2]Institute of Ocean Engineering, Shenzhen International Graduate School, Tsinghua University, Shenzhen, Guangdong 518055, China.

[3]School of Natural Sciences, Macquarie University, Sydney, NSW, Australia.

[4]U.S. Geological Survey, Wetland and Aquatic Research Center, 700 Cajundome Blvd Lafayette, LA 70506, USA.

[5]Silvestrum Climate Associates LLC, 1 Crescent Ave, Sausalito CA 94965, USA.

[6]Institute of Marine Ecology and Environment and Hainan International Blue Carbon Research Center, Hainan Academy of Environmental Sciences, Haikou, Hainan 570100, China.

*Corresponding author: lingh@tsinghua.edu.cn; wli2019@tsinghua.edu.cn; dingyali@mail.tsinghua.edu.

Supplementary Note 1. Influence of climate factors and restoration pathways on carbon accumulation

A linear mixed model was used to test the influence of restoration pathway and casual climate factors on the aboveground and belowground biomass carbon accumulation. We included age, restoration pathway, mean annual temperature (MAT), mean annual precipitation (MAP), and their interactions as fixed factors, with restoration region as a random factor. Moreover, ages of sampling plots were divided into 5-year age classes, but were grouped for 20–40 years due to the limited sample size. In each age group, mangrove restoration sites were further classified into three groups with different precipitation levels of 500–1500, 1500–2500 and 2500–3500 mm yr^{-1} of MAP. In each age group, the significance level of difference among different precipitation levels and restoration pathways was assessed by the Kruskal–Wallis test.

Results of the linear mixed model show that both restoration pathways and MAP can influence the aboveground biomass carbon accumulation over time (log (Age) × restoration pathways, $P \leq 0.05$; log (Age) × MAP, $P \leq 0.001$, Supplementary Table 2), while belowground biomass carbon is only influenced by MAP. For both restoration pathways, greater aboveground biomass carbon accumulation always occurs in regions with higher amount of precipitation (except for afforestation sites with MAP of 2500–3500 mm yr^{-1}). Meanwhile, within each MAP interval, mangrove reforestation achieved a greater aboveground biomass carbon accumulation than afforestation (Supplementary Fig. 4). In short, MAP and restoration pathways jointly controlled the carbon sequestration of aboveground biomass during mangrove restoration at the global scale.

In our database, there is no significant difference of MAP between mangrove reforestation and afforestation sites (Wilcoxon test, W=22112, $P>0.05$), although MAP in the reforestation sites is more evenly distributed, and MAP in the afforestation sites mainly concentrates ~2000 mm yr^{-1} (Supplementary Fig. 4). The interaction between MAP and restoration pathways (log (Age) × restoration pathways × MAP, $P>0.05$; Supplementary Table 2) is non-significant, suggesting that carbon accumulation during mangrove reforestation and afforestation was influenced by MAP to a similar extent.

Supplementary Note 2. Influence of sediment salinity on biomass portioning pattern

The sediment properties could not only influence the biomass growth, but also the biomass allocation patterns[1-3]. As revealed by the optimal allocation theory, plants prefer to regulate their biomass allocation to the organs acquiring the most limiting resources for maximizing growth[1]. Our results detected a significant positive relationship between sediment porewater salinity and the ratio of BGC to AGC (BGC/AGC, Supplementary Fig. 6), indicating the higher salinity condition may drive more biomass allocation belowground, as noted in natural field studies[4,5]. With salinity stress increasing, hydraulic conductivity as well as water/nutrient uptake rate in mangroves were diminished as xylem tissue became narrower and denser to avoid cavitation[2]. Greater biomass portioning to roots, therefore, may compensate for the limited water and nutrient absorption by individual mangrove trees[3,6]. Furthermore, the interaction of higher porewater salinity with lower nutrient content at mangrove afforestation sites could promote belowground biomass carbon accumulation, which might partially explain why afforestation sites exhibited a lower carbon accumulation rate among aboveground components than reforestation, whilst the rate of belowground carbon increments were similar (Fig. 2).

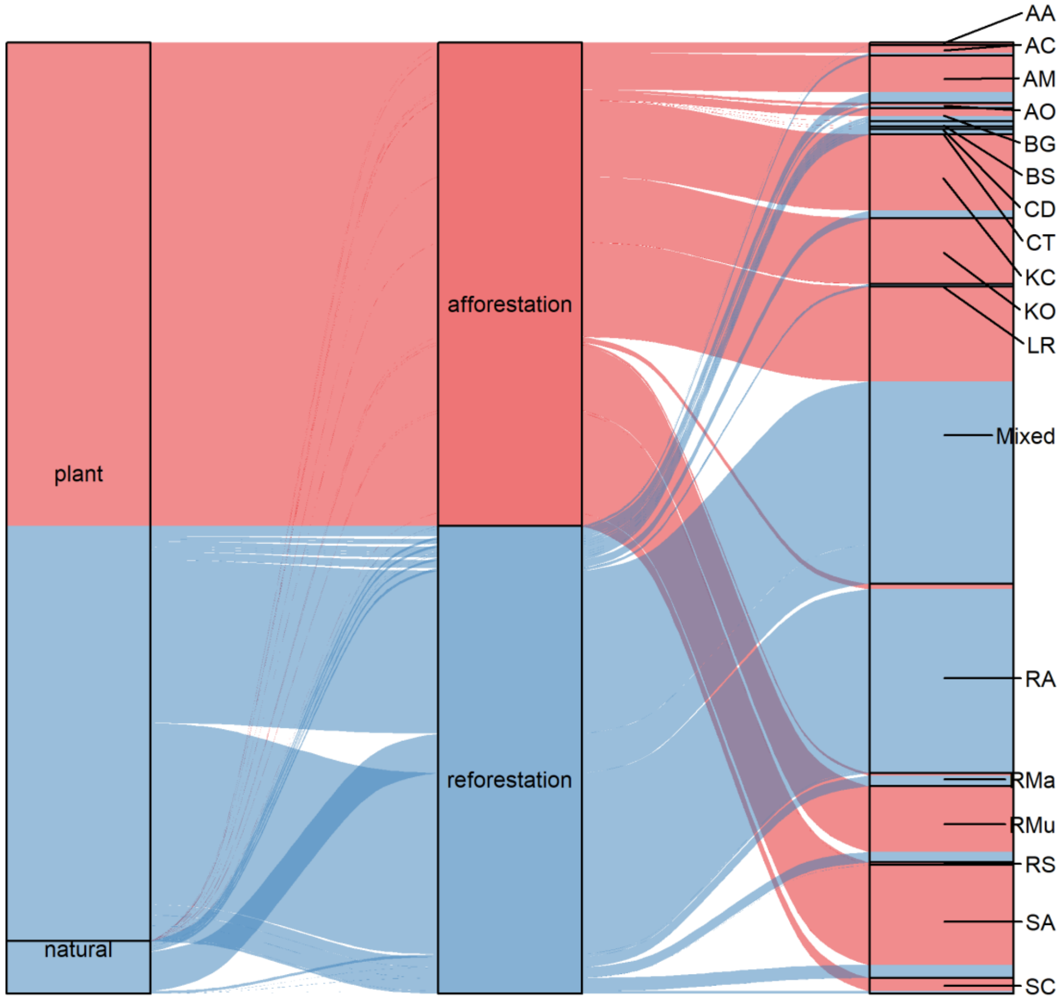

Fig. 1 Species and methods used by mangrove afforestation and reforestation projects in our analysis. natural: restoration by natural regeneration; plant: restoration by planting mangroves. Mixed: mixed-species used during mangrove restoration. AA: *Avicennia alba*; AC: *Aegiceras corniculatum*; AM: *Avicennia marina*; AO: *Avicennia officinalis*; BG: *Bruguiera gymnorrhiza*; BS: *Bruguiera sexangula*; CD: *Ceriops decandra*; CT: *Ceriops tagal*; KC: *Kandelia candel*; KO: *Kandelia obovata*; LR: *Laguncularia racemosa*; RA: *Rhizophora apiculata*; RMa: *Rhizophora mangle*; RMu: *Rhizophora mucronata*; RS: *Rhizophora stylosa*; SA: *Sonneratia apetala*; SC: *Sonneratia caseolaris*.

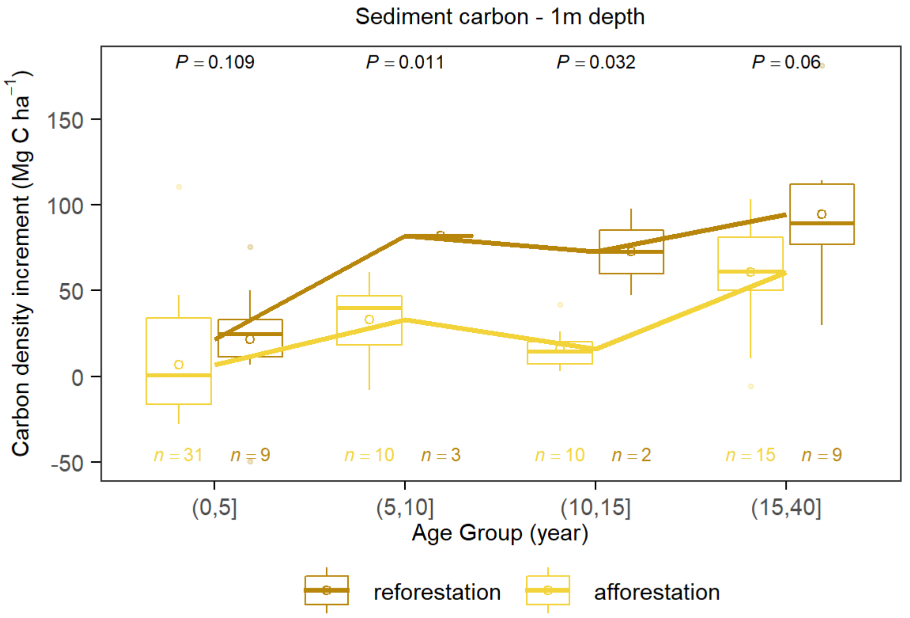

Fig. 2 Sediment carbon increments from the onset of mangrove reforestation and afforestation. Sediment carbon density increments are calculated as the difference between each restoration site and its paired control site. The x-axis represents different age groups (0–5, 5–10, 10–15, 15–40 years). For the box plot, the center line and the top and bottom of the box represent the median and the interquartile range (25th and 75th percentile). The whiskers represent the minimum and maximum limits, and the outliers are represented by dots. Mean carbon density in each age group and each MAP group is represented by a circle and connected by a line. Sample size (*n*) of each group is showed at the bottom. Significance level of difference between reforestation and afforestation within each age group is calculated by the Wilcoxon two-sided test.

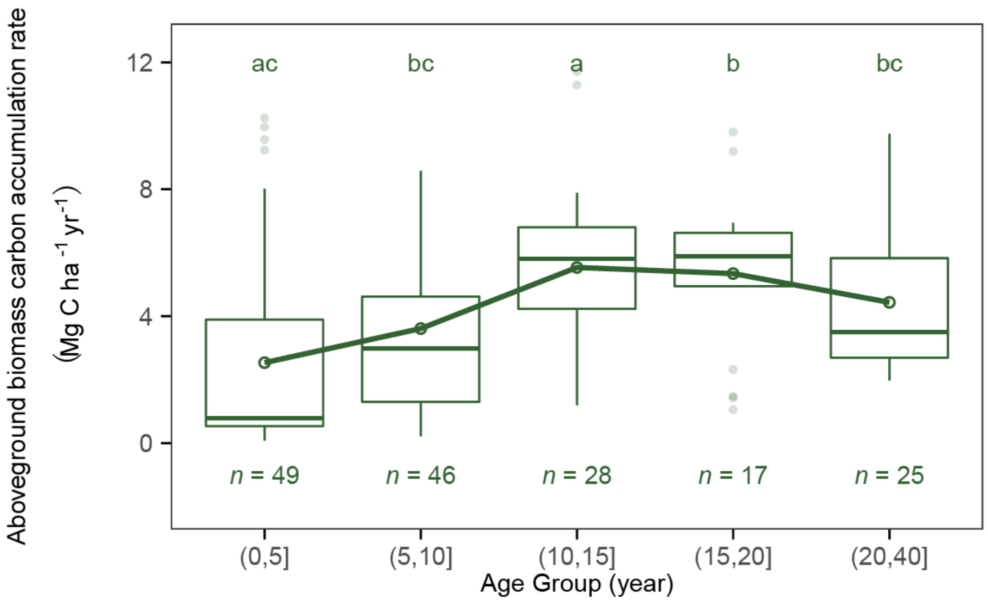

Fig. 3 Average aboveground biomass carbon accumulation rate during mangrove reforestation for different age groups. Average aboveground biomass carbon accumulation rate is calculated by the biomass carbon density at each age divided by the age. The x-axis represents different age groups (0–5, 5–10, 10–15, 15–20, and 20–40 years). For each box plot, individual data points are shown as circles, the center line and the top and bottom of the box represent the median and the interquartile range (25th and 75th percentile). The whiskers represent the minimum and maximum limits. Outliers are represented by dots. Mean carbon density in each age group is represented by a circle and connected by a line. Sample size (*n*) of each group is shown at the bottom of each boxplot. Significance level of difference among age groups is calculated by the Kruskal–Wallis test combined with the Bonferroni adjusted post hoc Dunn test. Groups sharing the same letter are not significantly different at level of 0.05.

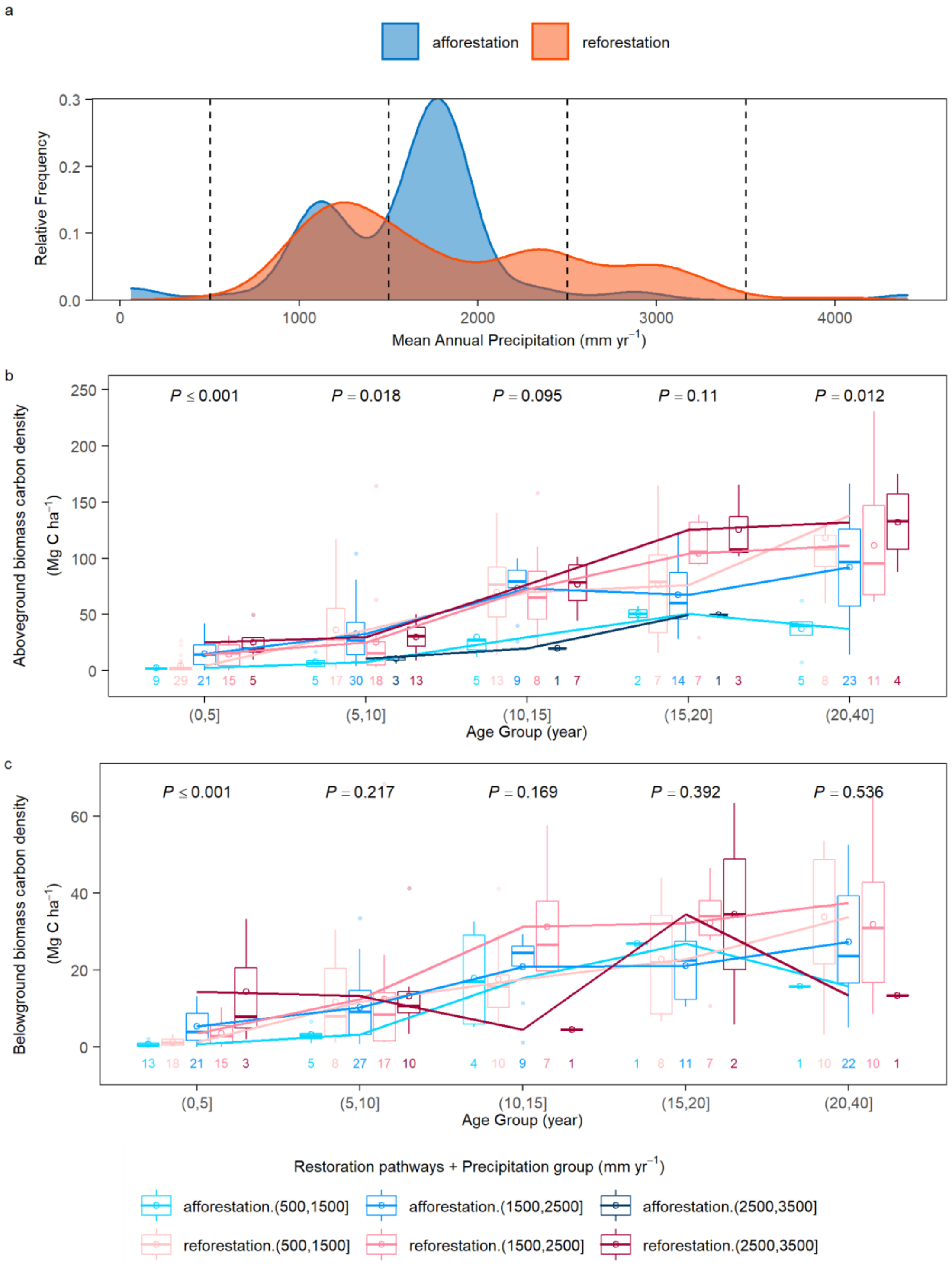

Fig. 4 Comparison of carbon densities between mangrove reforestation and afforestation sites in different precipitation intervals over a 40-year period. a, distribution of mean annual precipitation (MAP) in mangrove reforestation and afforestation sites. Vertical lines from left to right represents MAP levels of 500, 1500, 2500 and 3500 mm yr^{-1}, which are used as the boundary values for different precipitation group. Aboveground (**b**) and belowground (**c**) carbon density changes

with mangrove ages (x-axis) for each precipitation group (different colors). The x-axis represents different age groups (0–5, 5–10, 10–15, 15–20, and 20–40 years). For the box plot, the center line and the top and bottom of the box represent the median and the interquartile range (25th and 75th percentile). The whiskers represent the minimum and maximum limits, and the outliers are represented by dots. Mean carbon density in each age group and each MAP group is represented by a circle and connected by a line. Sample size (n) of each group is showed at the bottom. Significance level of difference between reforestation and afforestation within each age group is calculated by the Kruskal–Wallis test.

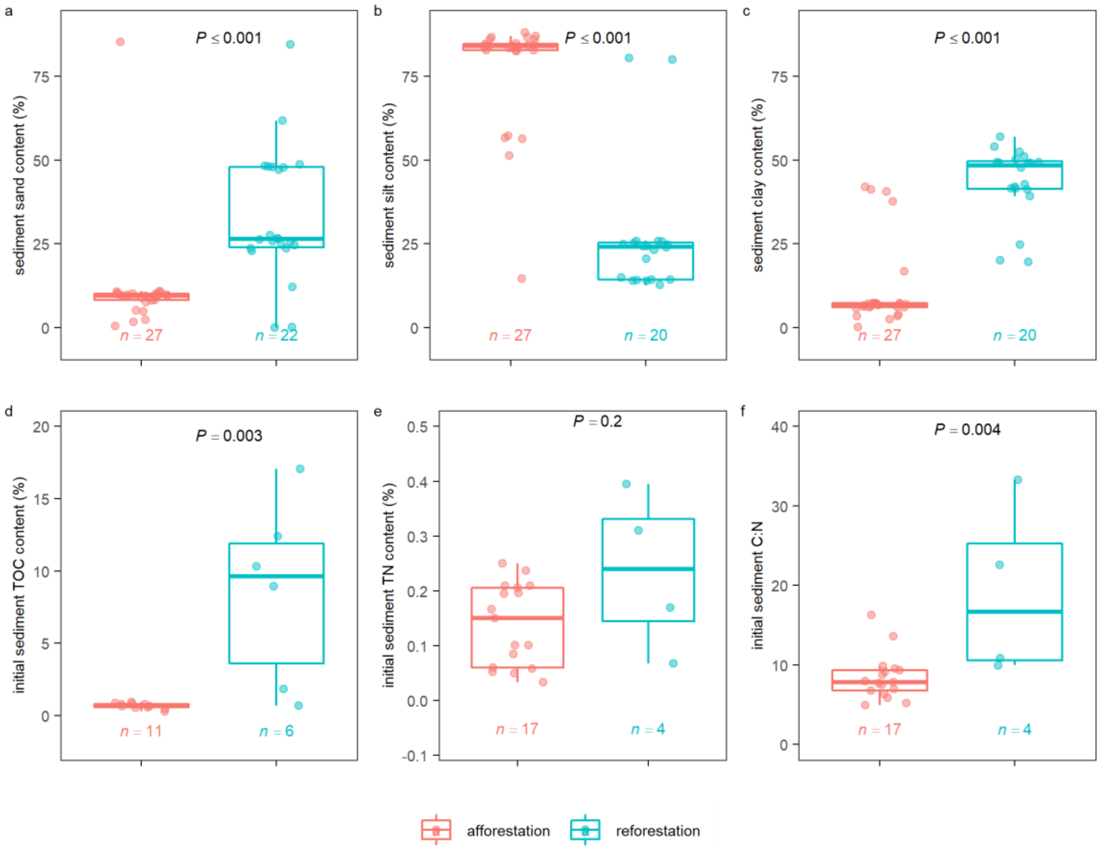

Fig. 5 Comparison of sediment particle size fraction and initial conditions associated with mangrove reforestation versus afforestation. C: carbon; TOC: total organic carbon; TN: total nitrogen; C:N: ratio of TOC content to TN content. For each box plot, individual data points are shown as circles. The center line and the top and bottom of the box represent the median and the interquartile range (25th percentile and 75th percentile). The whiskers represent the minimum and maximum limits. Sample size (*n*) of each group is shown at the top of each boxplot. Significance level of difference between reforestation and afforestation for each parameter is calculated by the Wilcoxon two-sided test.

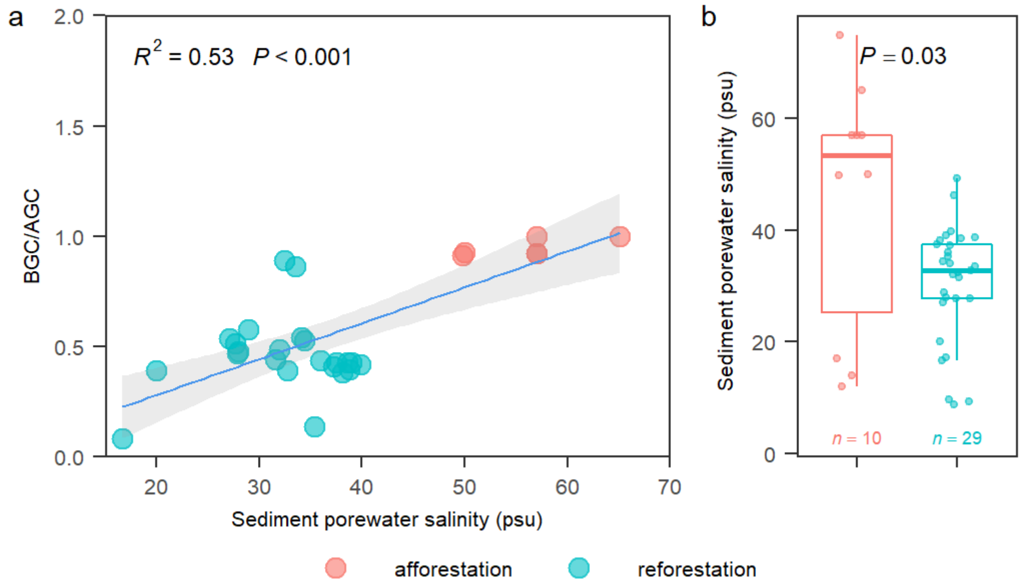

Fig. 6 Sediment porewater salinity and its relationship to the ratio of belowground biomass carbon to aboveground biomass carbon (BGC/AGC) from mangrove afforestation and reforestation projects in our analysis. a, BGC/AGC ratio and sediment porewater salinity as detected by the ordinary least squares regression. The grey area indicates 95% confidence interval. psu refers to practical salinity units. **b**, Sediment porewater salinity for reforestation and afforestation sites. For each box plot, individual data points are shown as circles. The center line and the top and bottom of the box represent the median and the interquartile range (25th percentile and 75th percentile). The whiskers represent the minimum and maximum limits. Sample size (*n*) of each group is shown at the top of each boxplot. Significance level of difference between reforestation and afforestation is calculated by the Wilcoxon two-sided test.

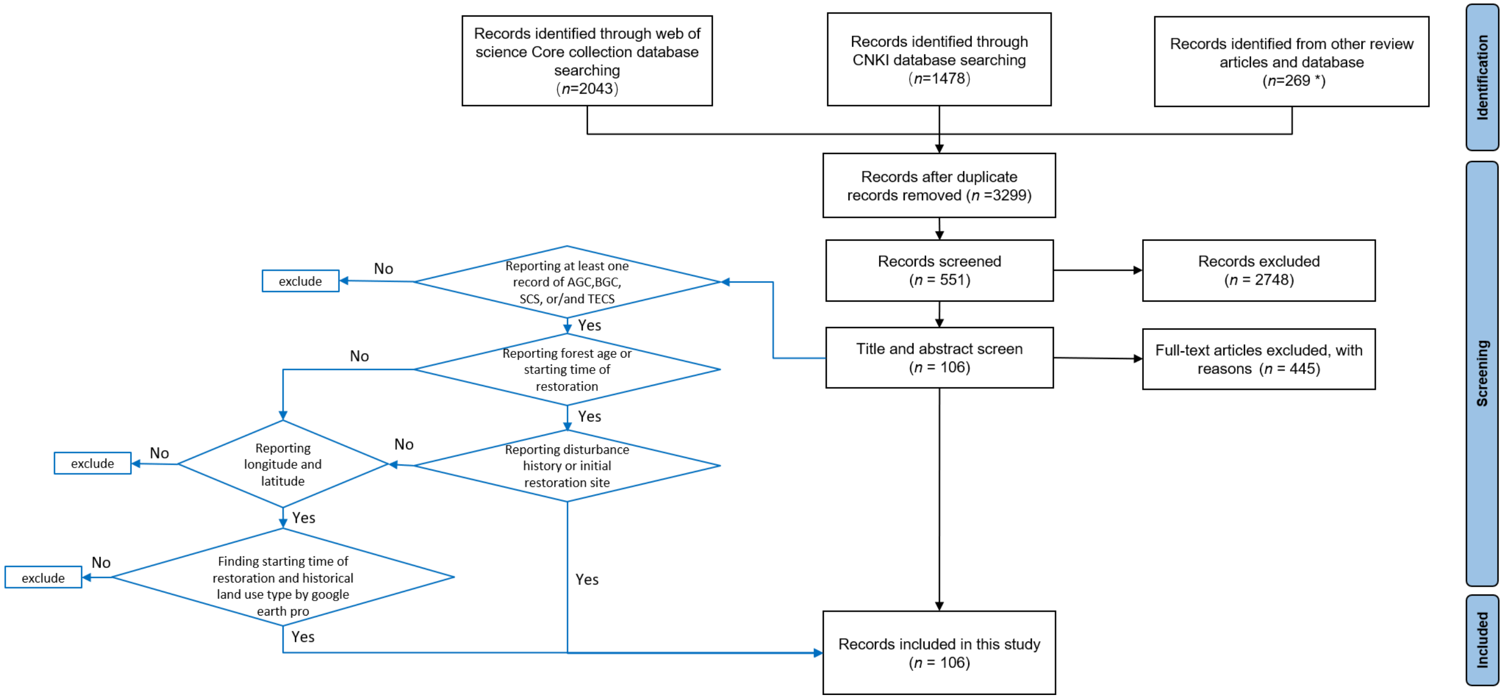

Fig. 7 PRISMA flow chart used to search for relevant articles. Blue boxes (diamonds) represent required criteria. * 269 records in review articles and databases included 11 records in Meng, et al. [7], 54 records in Xiong, et al. [8], 20 records in Chen, et al. [9], 19 records in Sasmito, et al. [10], 26 records in Bernal, et al. [11], 6 records in Su, et al. [12] and 133 records using "ecological restoration" in The Sustainable Wetlands Adaptation and Mitigation Program (SWAMP) database (https://www2.cifor.org/swamp/database/database-management/). AGC: aboveground biomass carbon, BGC: belowground biomass carbon, SCS: sediment carbon, TECS: total ecosystem carbon, CNKI: China National Knowledge Infrastructure.

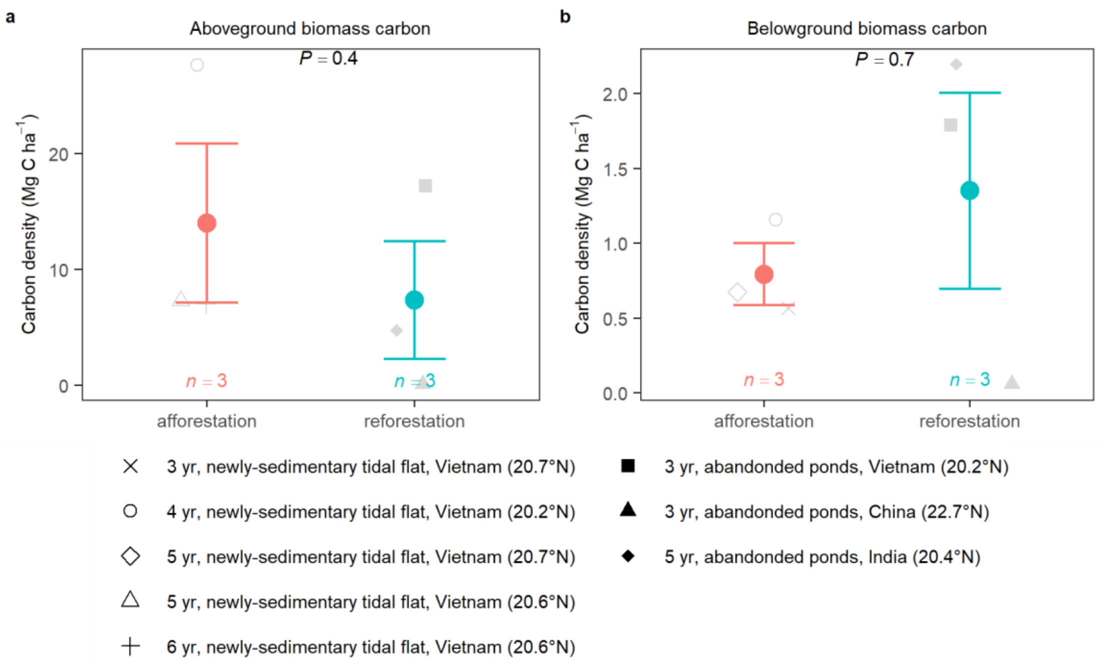

Fig. 8 Comparison of *Kandelia spp.* aboveground and belowground biomass carbon between afforestation in newly-sedimentary flat and reforestation in its nearby abandoned ponds. Sample size (*n*) of each group is shown at the top of each boxplot. Difference between reforestation and afforestation was calculated by the Wilcoxon two-sided test. Values are means ± standard error.

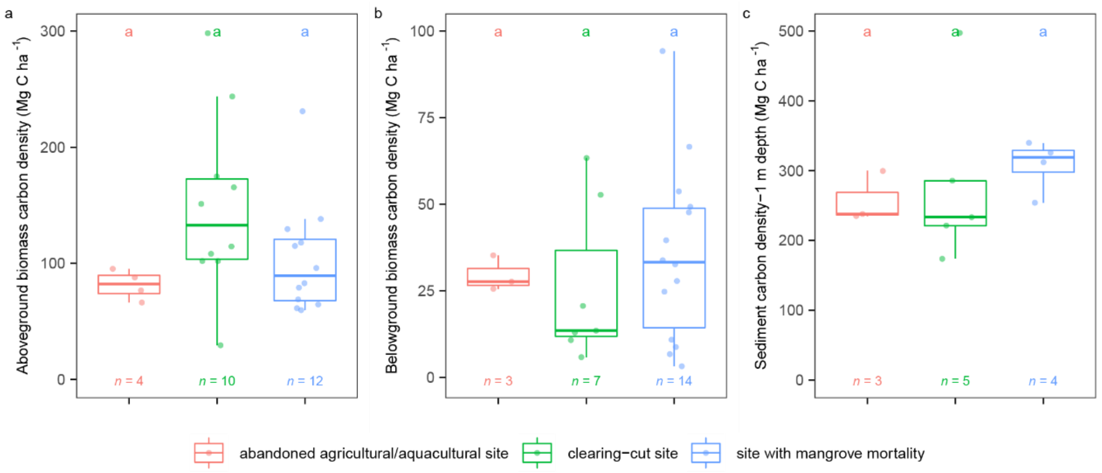

Fig. 9 Comparison of mangrove carbon densities after 15-40 years of restoration among sites with different causes of degradation/deforestation. For each box plot, individual data points are shown as circles. The center line and the top and bottom of the box represent the median and the interquartile range (25th percentile and 75th percentile). The whiskers represent the minimum and maximum limits. Sample size (*n*) of each group is showed at the bottom. Significance level of difference among age groups is calculated by the Kruskal–Wallis test combined with the Bonferroni adjusted post hoc Dunn test. Groups sharing the same letter were not significantly different at level of 0.05.

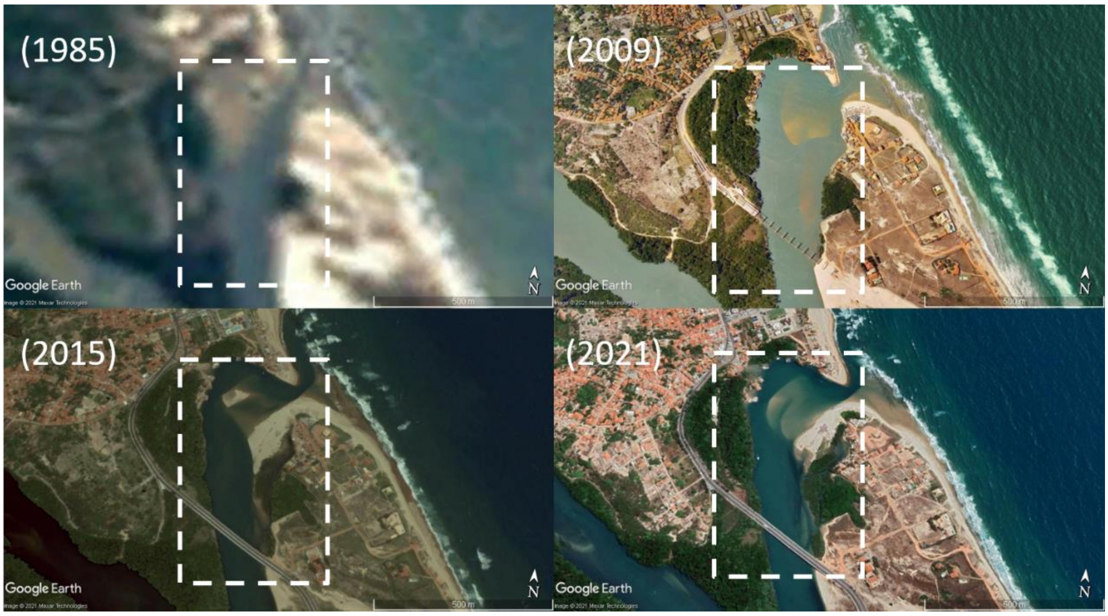

Fig. 10 An example for inference of prior land use type and initial restoration time by remote sensing. The number in the upper left conner represents the year when the satellite image was taken. Jimenez, et al. [13] provided the location of sampling plot and the restoration age in their Fig. 1, and the satellite image revealed that the mangrove was restored in the newly accreted mudflat due to the construction of a highway.

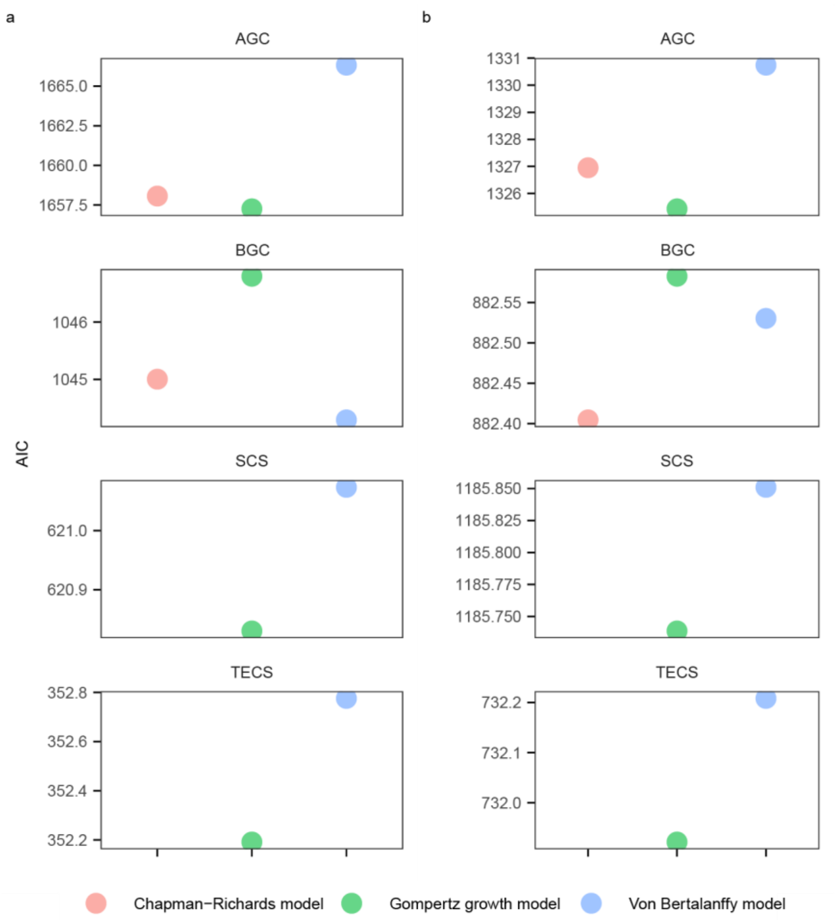

Fig. 11 Akaike information criterion (AIC) of models that mimic accumulation trajectories in the four mangrove carbon pools reviewed for (a) reforestation and (b) afforestation. AGC: aboveground biomass carbon; BGC: belowground biomass carbon; SCS: sediment carbon to 1-m depth; TECS: total ecosystem carbon. Different colors represent different models. Models with lower AIC had a better fit.

Table 1. Influences of restoration type on carbon stocks over time as examined by linear mixed statistical models. AGC: aboveground biomass carbon; BGC: belowground biomass carbon; SCS: sediment carbon; TECS: total ecosystem carbon. Restoration pathways: reforestation and afforestation; Log (Age): log-transformed mangrove age. AIC: Akaike information criterion. A lower AIC value indicates a better model fit. ANOVA was used to evaluate the significance of each variable. Significant variables ($P \leq 0.05$) are shown in bold.

Carbon pool	Factors	*P*-value	AIC
AGC	**Age**	**<0.001**	2931.48
	Restoration pathways	0.14	
	**Age × Restoration pathways**	**<0.001**	
	**Log (Age)**	**<0.001**	2927.74
	**Restoration pathways**	**0.01**	
	**Log (Age) × Restoration pathways**	**<0.001**	
BGC	**Age**	**<0.001**	1885.89
	Restoration pathways	0.89	
	Age × Restoration pathways	0.44	
	**Log (Age)**	**<0.001**	1881.24
	Restoration pathways	0.34	
	Log (Age) × Restoration pathways	0.12	
SCS	Age	0.06	1394.22
	**Restoration pathways**	**0.003**	
	Age × Restoration pathways	0.30	
	**Log (Age)**	**0.04**	1377.13
	Restoration pathways	0.81	
	**Log (Age) × Restoration pathways**	**0.013**	
TECS	**Age**	**0.003**	1002.65
	**Restoration pathways**	**0.009**	
	Age × Restoration pathways	0.25	
	**Log (Age)**	**<0.001**	981.61
	Restoration pathways	0.68	
	**Log (Age) × Restoration pathways**	**0.03**	

Table 2. Impacts of climate factors and restoration types on carbon densities over time examined by linear mixed models. AGC: aboveground biomass carbon; BGC: belowground biomass carbon. Restoration pathways: reforestation and afforestation; MAT: mean annual temperature; MAP: mean annual precipitation. AIC: Akaike information criterion. AIC-log: model with log-transformed age; AIC-original: model with age. A lower AIC value indicates a better model fit. ANOVA was used to evaluate the significance of each variable. Significant variables (*P*≤0.05) are shown in bold.

Carbon pool	Variable	*P*-value	AIC-log	AIC-original
AGC	**Log (Age)**	**<0.001**	2954.33	2939.29
	Restoration pathways	0.68		
	MAT	0.37		
	**MAP**	**0.03**		
	**Log (Age) × Restoration pathways**	**0.02**		
	Log (Age) × MAT	0.72		
	**Log (Age) × MAP**	**<0.001**		
	Restoration pathways × MAP	0.98		
	Restoration pathways × MAT	0.11		
	Log (Age) × Restoration pathways × MAP	0.81		
	Log (Age) × Restoration pathways × MAT	0.07		
BGC	**Log (Age)**	**<0.001**	1927.00	1908.68
	Restoration pathways	0.77		
	MAT	0.33		
	**MAP**	**0.001**		
	Log (Age) × Restoration pathways	0.36		
	Log (Age) × MAT	0.69		
	**Log (Age) × MAP**	**0.04**		
	**Restoration pathways × MAP**	**0.04**		
	Restoration pathways × MAT	0.91		
	Log (Age) × Restoration pathways × MAP	0.05		
	Log (Age) × Restoration pathways × MAT	0.14		

Table 3. Keywords used during our literature search. CNKI = China National Knowledge Infrastructure.

Platform	Keywords
Web of science	TS = ("biomass" OR "carbon" OR "soil" OR "sediment") AND TS = ("age" OR "chronology" OR "reforest*" OR "restoration" OR "rehabilit*" OR "artificial" OR "plantation" OR "regenerat*") AND TS = ("mangrove")
CNKI	TS=(Mangrove) AND TS = (biomass + carbon + soil + sediment) AND TS= (Growth + Recovery + Artificial + Planting + Expansion + Repair + Regeneration + Secondary Growth + Forest Age) in Chinese

Table 4. Biomass allometric equations used to calculate belowground biomass for later conversion to carbon density. B: belowground biomass (kg); DBH: diameter at breast height (cm in RA and Rmu; m in KC/KO/AC/AM); H: tree height (m); D: wood density (g cm^{-3}, http://db.world agroforestry.org/). RA: *Rhizophora apiculata*; RMu: *Rhizophora mucronata*; KC: *Kandelia candel*; KO: *Kandelia obovata*; AC: *Aegiceras corniculatum*; AM: *Avicennia marina*.

	**Belowground biomass allometric equations**	**Wood density/g cm^{-3}**	**Reference**	**Region**
RA	$B=0.199DBH^{0.899}D^{2.22}$	0.850	[14]	general
Rmu	$B=0.199DBH^{0.899}D^{2.22}$	0.820	[14]	
KC/KO	$lgB=2.433+0.990lg(DBH^2H)$	0.527	[15]	Shenzhen, China
AC	$lgB=0.967+0.303lg(DBH^2H)$	0.597	[15]	
AM	$lgB=1.361+0.615lg(DBH^2H)$	0.713	[15]	

Table 5. Parameters for the carbon accumulation models developed from mangrove reforestation and afforestation projects. Asym: asymptote for each carbon density pool as mangrove stands develop to maturity, b and c determine the slope and shape of the curve from origin to asymptote. RSE: residual standard error. Numbers in parentheses represent the 95% confidence intervals of parameter fit. AGC: aboveground biomass carbon, BGC: belowground biomass carbon, SCS: sediment carbon, TECS: total ecosystem carbon.

$C = Asym * e^{-bc^{Age}}$		**Asym (Mg C ha^{-1})**	**b**	**c**	**RSE**
Afforestation	AGC	89.82 (68.67, 110.96)	3.93 (1.79, 6.07)	0.87 (0.80, 0.93)	28.94
	BGC	40.30 (16.77, 63.83)	3.26 (2.33, 4.19)	0.91 (0.85, 0.97)	8.33
	SCS	185.29 (-175.99, 546.57)	0.69 (-1.19, 2.58)	0.96 (0.81, 1.12)	57.26
	TECS	229.17 (135.47,322.87)	1.13 (0.52, 1.74)	0.90 (0.77, 1.02)	77.55
Reforestation	AGC	128.23 (110.38, 146.08)	6.27 (2.71, 9.83)	0.83 (0.78, 0.89)	34.06
	BGC	39.29 (29.30, 49.28)	3.66 (1.57, 5.74)	0.87 (0.80, 0.94)	13.72
	SCS	323.72 (244.92, 402.51)	0.57 (0.15, 0.98)	0.86 (0.66, 1.07)	114.47
	TECS	435.8 (319.07, 552.53)	0.95 (0.09, 1.81)	0.87 (0.68, 1.06)	119.58

Table 6. Average proportion of mangrove loss drivers in each geographical zone calculated from Goldberg, et al. [16].

Column headings represent drivers of mangrove deforestation; NPC: nonproductive conversion.

Geographical zone	erosion	extreme climate	commodities	NPC	settlement
Australia and New Zealand	0.20	0.69	0.00	0.10	0.00
Caribbean	0.06	0.04	0.86	0.05	0.00
Central America	0.28	0.09	0.21	0.36	0.05
Eastern Africa	0.25	0.33	0.05	0.37	0.01
Melanesia & Micronesia & Polynesia	0.60	0.19	0.10	0.10	0.01
Middle Africa	0.36	0.35	0.02	0.26	0.01
Northern America	0.34	0.29	0.02	0.26	0.09
South America	0.54	0.24	0.13	0.08	0.00
South-Eastern Asia	0.19	0.03	0.72	0.04	0.03
Southern Africa	0.62	0.00	0.00	0.38	0.00
Southern Asia	0.56	0.08	0.09	0.21	0.05
Western Africa	0.25	0.18	0.01	0.46	0.11
Western Asia	0.28	0.40	0.07	0.24	0.01

Disclaimer: Any use of trade, firm, or product names is for descriptive purposes only and does not imply endorsement by the U.S. Government.

References

1 Bloom, A. J., Chapin Iii, F. S. & Mooney, H. A. Resource limitation in plants-an economic analogy. *Annual review of Ecology and Systematics* **16**, 363-392 (1985).

2 Reef, R. & Lovelock, C. E. Regulation of water balance in mangroves. *Annals of Botany* **115**, 385-395 (2015).

3 Vovides, A. G. *et al.* Morphological plasticity in mangrove trees: salinity-related changes in the allometry of *Avicennia germinans*. *Trees* **28**, 1413-1425 (2014).

4 Saintilan, N. Above and below-ground biomass of mangroves in a sub-tropical estuary. *Marine and Freshwater Research* **48**, 601-604 (1997).

5 Saintilan, N. Above-and below-ground biomasses of two species of mangrove on the Hawkesbury River estuary, New South Wales. *Marine and freshwater research* **48**, 147-152 (1997).

6 Ball, M. C. Salinity tolerance in the mangroves *Aegiceras corniculatum* and *Avicennia marina. I.* Water use in relation to growth, carbon partitioning, and salt balance. *Functional Plant Biology* **15**, 447-464 (1988).

7 Meng, Y. *et al.* Relationships between above- and below-ground carbon stocks in mangrove forests facilitate better estimation of total mangrove blue carbon. *Carbon Balance and Management* **16**, 8 (2021).

8 Xiong, Y., Liao, B. & Wang, F. Mangrove vegetation enhances soil carbon storage primarily through in situ inputs rather than increasing allochthonous sediments. *Marine pollution bulletin* **131**, 378-385 (2018).

9 Chen, S. *et al.* Higher soil organic carbon sequestration potential at a rehabilitated mangrove comprised of *Aegiceras corniculatum* compared to *Kandelia obovata*. *Science of The Total Environment* **752**, 142279 (2021).

10 Sasmito, S. D. *et al.* Effect of land-use and land-cover change on mangrove blue carbon: A systematic review. *Global Change Biology* **25**, 4291-4302 (2019).

11 Bernal, B., Murray, L. T. & Pearson, T. R. H. Global carbon dioxide removal rates

from forest landscape restoration activities. *Carbon Balance and Management* **13**, 22 (2018).

12 Su, J., Friess, D. A. & Gasparatos, A. A meta-analysis of the ecological and economic outcomes of mangrove restoration. *Nature Communications* **12**, 5050 (2021).

13 Jimenez, L. C., Queiroz, H. M., Otero, X. L., Nóbrega, G. N. & Ferreira, T. O. Soil organic matter responses to mangrove restoration: A replanting experience in northeast Brazil. *International Journal of Environmental Research and Public Health* **18**, 8981 (2021).

14 Komiyama, A., Ong, J. E. & Poungparn, S. Allometry, biomass, and productivity of mangrove forests: A review. *Aquatic Botany* **89**, 128-137 (2008).

15 Tam, N. F. Y., Wong, Y. S., Lan, C. Y. & Chen, G. Z. Community structure and standing crop biomass of a mangrove forest in Futian Nature Reserve, Shenzhen, China. *Hydrobiologia* **295**, 193-201 (1995).

16 Goldberg, L., Lagomasino, D., Thomas, N. & Fatoyinbo, T. Global declines in human-driven mangrove loss. *Global Change Biology* **26**, 5844-5855 (2020).
