# Supplementary material for: Mangrove reforestation provides greater blue carbon benefit than afforestation for mitigating global climate change
Source: Nat Commun. 2023 Feb 10;14:756. doi: 10.1038/s41467-023-36477-1 (PMC9918466; doi:10.1038/s41467-023-36477-1)
Supplement: Supplementary file 2 — Reporting Summary [file 41467_2023_36477_MOESM2_ESM.pdf]

nature portfolio

Corresponding author(s): Guanghui Lin

Last updated by author(s): Jan 20, 2023

# Reporting Summary

Nature Portfolio wishes to improve the reproducibility of the work that we publish. This form provides structure for consistency and transparency in reporting. For further information on Nature Portfolio policies, see our Editorial Policies and the Editorial Policy Checklist.

## Statistics

For all statistical analyses, confirm that the following items are present in the figure legend, table legend, main text, or Methods section.

| n/a | Confirmed | |
|---|---|---|
| ☐ | ☒ | The exact sample size ($n$) for each experimental group/condition, given as a discrete number and unit of measurement |
| ☐ | ☒ | A statement on whether measurements were taken from distinct samples or whether the same sample was measured repeatedly |
| ☐ | ☒ | The statistical test(s) used AND whether they are one- or two-sided<br>*Only common tests should be described solely by name; describe more complex techniques in the Methods section.* |
| ☐ | ☒ | A description of all covariates tested |
| ☐ | ☒ | A description of any assumptions or corrections, such as tests of normality and adjustment for multiple comparisons |
| ☐ | ☒ | A full description of the statistical parameters including central tendency (e.g. means) or other basic estimates (e.g. regression coefficient) AND variation (e.g. standard deviation) or associated estimates of uncertainty (e.g. confidence intervals) |
| ☐ | ☒ | For null hypothesis testing, the test statistic (e.g. $F$, $t$, $r$) with confidence intervals, effect sizes, degrees of freedom and $P$ value noted<br>*Give P values as exact values whenever suitable.* |
| ☒ | ☐ | For Bayesian analysis, information on the choice of priors and Markov chain Monte Carlo settings |
| ☒ | ☐ | For hierarchical and complex designs, identification of the appropriate level for tests and full reporting of outcomes |
| ☒ | ☐ | Estimates of effect sizes (e.g. Cohen's $d$, Pearson's $r$), indicating how they were calculated |

*Our web collection on statistics for biologists contains articles on many of the points above.*

## Software and code

Policy information about availability of computer code

| | |
|---|---|
| Data collection | No field data was collected in this study |
| Data analysis | GetData Graph Digitizer version 2.24 was used during data collection. All data analysis was performed in R version 4.0.4, We used lme4 (v 1.1.29), emmeans (v 1.7.5), nlme (v 3.1.157), nlstools (v 2.0.0), car (v 3.0.13), stats (v 4.2.0), FSA (v 0.9.3), rcompanion (v 2.4.18), ggplot2(v 3.3.6), dplyr (version 1.0.9), tidyr (v 1.2.0), rstatix (v 0.7.0), ggpmisc (v 0.4.6), ggpubr (v 0.4.0), ggrepel (v 0.9.1), ggalluvial (v 0.12.3), reshape2 (v 1.4.4), introdataviz (v 0.0.0.9003), sf (v 1.0.7), rnaturalearth (v 0.1.0), rnaturalearthdata (v 0.1.0), nlraa (v 1.2), agricolae (v 1.3.5) packages. All data analysis and figure visualization are available on https://doi.org/10.5281/zenodo.7556422. |

For manuscripts utilizing custom algorithms or software that are central to the research but not yet described in published literature, software must be made available to editors and reviewers. We strongly encourage code deposition in a community repository (e.g. GitHub). See the Nature Portfolio guidelines for submitting code & software for further information.

## Data

Policy information about availability of data

All manuscripts must include a data availability statement. This statement should provide the following information, where applicable:

- Accession codes, unique identifiers, or web links for publicly available datasets
- A description of any restrictions on data availability
- For clinical datasets or third party data, please ensure that the statement adheres to our policy

All data collected through literature search are available at https://figshare.com/s/8c15165a6c9a2732574f; data supporting Fig. S8 are available at https://

figshare.com/s/adf4786133469d39fc8b; mangrove area dataset (Global mangrove watch 1996 and 2016) is available at https://data.unep-wcmc.org/datasets/45; assumption and calculation process of climate mitigation potential from global mangrove reforestation under biophysical constraint is available via https://figshare.com/s/0592721c88b9a6843166; mangrove losing area in each country and its exclusive economic zone are available via https://figshare.com/s/f5b1e6afd098d0fbe94c; WorldClim2.0 dataset are available via https://www.worldclim.org/data/worldclim21.html; The union of world country boundaries and its exclusive economic zone are avaiable via https://www.marineregions.org/downloads.php. Source data of figures and tables are provided in the Source Data file with this paper.

## Human research participants

Policy information about studies involving human research participants and Sex and Gender in Research.

| | |
|---|---|
| Reporting on sex and gender | NA |
| Population characteristics | NA |
| Recruitment | NA |
| Ethics oversight | NA |

Note that full information on the approval of the study protocol must also be provided in the manuscript.

# Field-specific reporting

Please select the one below that is the best fit for your research. If you are not sure, read the appropriate sections before making your selection.

☐ Life sciences ☐ Behavioural & social sciences ☒ Ecological, evolutionary & environmental sciences

For a reference copy of the document with all sections, see nature.com/documents/nr-reporting-summary-flat.pdf

# Ecological, evolutionary & environmental sciences study design

All studies must disclose on these points even when the disclosure is negative.

**Study description**

Blue carbon density data during mangrove restoration were collected via literature searches on Web of Science and China National Knowledge Infrastructure (CNKI) platforms. Historical land-use type, sediment properties and mangrove forest age were also collected. Mangrove restoration could be divided into two categories – reforestation and afforestation – by whether mangroves previously existed in that location. Difference of carbon accumulation potential and its environmental driving forces were compared between mangrove reforestation and afforestation. Global carbon sequestration potential of mangrove reforestation action was also evaluated by using mangrove deforestation dataset and simulating carbon accumulation curves of mangrove carbon pools under several future mangrove restoration scenarios.

**Research sample**

We compiled a global carbon stock dataset of available mangrove restoration sites. We collected data from these regions including carbon density of four carbon pools (aboveground biomass, belowground biomass, sediment and total ecosystem carbon), forest age, historical land use type, restoration pathway (planting/natural expansion), sediment properties (including sediment carbon concentration, nitrogen concentration, pH, porewater salinity, particle size distribution), species and climate factors (Mean annual precipitation and temperature).

**Sampling strategy**

We collected data from four step procedures, (1) carbon stock data of at least one of the four mangrove carbon pools (i.e., aboveground biomass, belowground biomass, sediment organic carbon, or total ecosystem carbon) must be provided; (2) forest age or the starting date of the restoration action must be provided; (3) a description of prior land use was required; (4) if a prior land use type was not provided, we only included region whose historical land use type could be told by satellite images.

For studies that reported sediment carbon stock to < 1 m depth, we assumed that its organic sediment layer was deeper than 1 m and that the carbon density of the unmeasured depth was the same as that of the deepest measured layer. Studies that only measured surface sediment carbon (< 20 cm) was not incorporated in our dataset.

**Data collection**

All the data were compiled from original research literatures searched from Web of Science and CNKI. We also included data from some peer-reviewed datasets and online datasets (The Sustainable Wetlands Adaptation and Mitigation Program (SWAMP) database, https://www2.cifor.org/ swamp).

We extracted mangrove aboveground living biomass carbon, belowground living biomass carbon, sediment carbon, and total ecosystem carbon density from the manuscripts or figures with the application of GetData Graph Digitizer. If biomass carbon pools were not provided, its biomass could also be calculated by trunk diameter at breast height (dbh, ~1.3 m) and tree height using species-specific or common allometric equations. Sediment carbon stock could also be calculated by multiplying sediment organic carbon content by bulk density, integrated over depth (cm).

Climate factors of each restoration sites were extracted from the WorldClim Bioclimatic variables for WorldClim2.0 dataset58 and averaged within 1 km buffer.

| | |
|---|---|
| Timing and spatial scale | Our dataset includes data from as many years as all the key data we needed (i.e., forest age, historical land use type, carbon density for at least one of the four carbon pools) were provided in the mangrove restoring sites. The spatial scale of our datasets is tropical and subtropical coastal areas where mangrove restoration occurred. |
| Data exclusions | Data were excluded using the criteria stated above. Literatures not providing historical land use type, forest age and/or relevant coordinate information were excluded. Studies that only measured surface sediment carbon (< 20 cm) was not in our dataset. We also excluded an article reporting outliers whose carbon density was two orders of magnitude higher than in other regions. |
| Reproducibility | The entire analysis can be reproduced following the code available at https://doi.org/10.5281/zenodo.7554762. |
| Randomization | Randomization was not relevant because no new data were collected. |
| Blinding | Blinding was not relevant because no new data were collected. |

Did the study involve field work? ☐ Yes ☒ No

# Reporting for specific materials, systems and methods

We require information from authors about some types of materials, experimental systems and methods used in many studies. Here, indicate whether each material, system or method listed is relevant to your study. If you are not sure if a list item applies to your research, read the appropriate section before selecting a response.

## Materials & experimental systems

| n/a | Involved in the study | |
|---|---|---|
| ☒ | ☐ | Antibodies |
| ☒ | ☐ | Eukaryotic cell lines |
| ☒ | ☐ | Palaeontology and archaeology |
| ☒ | ☐ | Animals and other organisms |
| ☒ | ☐ | Clinical data |
| ☒ | ☐ | Dual use research of concern |

## Methods

| n/a | Involved in the study | |
|---|---|---|
| ☒ | ☐ | ChIP-seq |
| ☒ | ☐ | Flow cytometry |
| ☒ | ☐ | MRI-based neuroimaging |
